# Mode of birth and development of maternal postnatal post‐traumatic stress disorder: A mixed‐methods systematic review and meta‐analysis

**DOI:** 10.1111/birt.12649

**Published:** 2022-05-13

**Authors:** Jemima Carter, Debra Bick, Daniel Gallacher, Yan‐Shing Chang

**Affiliations:** ^1^ Faculty of Life Sciences and Medicine King's College London London UK; ^2^ St Richard's Hospital Chichester UK; ^3^ Warwick Medical School University of Warwick Warwick UK; ^4^ Florence Nightingale Faculty of Nursing, Midwifery and Palliative Care King's College London London UK

**Keywords:** child birth, meta‐analysis, mixed‐methods, mode of birth, postpartum, post‐ traumatic stress disorder, systematic review

## Abstract

**Background:**

Post‐traumatic stress disorder (PTSD) affects approximately 3% of women in the postnatal period, but less is known about risk factors for PTSD than other postnatal mental illnesses. This review aimed to analyze the literature on the impact of mode of birth on postnatal PTSD.

**Methods:**

Searches were undertaken of CINAHL, the Cochrane Library, MEDLINE, PsycINFO, and Scopus for studies investigating the link between mode of birth and postnatal PTSD in high‐resource countries from January 1990 to February 2021. Quantitative and qualitative data were collected and synthesized. Meta‐analysis was performed with four of the studies, and the rest were analyzed narratively.

**Results:**

Twelve quantitative studies, presenting data on 5567 women, and two qualitative studies, with 92 women, were included in the review. Most studies found a significant relationship between mode of birth and maternal PTSD symptoms. Meta‐analysis found cesarean birth was more closely associated with PTSD than vaginal delivery (VD) (*P* = 0.005), emergency cesarean birth (EmCB) more than elective cesarean birth (ElCB) (*P* < 0.001), instrumental vaginal delivery (IVD) more than spontaneous vaginal delivery (SVD) (*P* < 0.001), and EmCB more than SVD (*P* < 0.001). Women who developed PTSD after EmCB felt less in control and less supported than those who did not develop it after the same procedure. Request for repeat ElCB appeared more common among women with pre‐existing postnatal PTSD, but this may subsequently leave them feeling dissatisfied and their fears of childbirth unresolved.

**Conclusions:**

Modes of birth involving emergency intervention may be risk factors for the development of postnatal PTSD. Ensuring that women feel supported and in control during emergency obstetric interventions may mediate against this risk.

## 1 INTRODUCTION

Post‐traumatic stress disorder (PTSD) is a psychiatric condition whereby individuals suffer flashbacks, anxiety, and avoidance after exposure to a traumatic event. Given that childbirth is usually a positive and life‐affirming event, only recently did the scientific community begin to recognize that giving birth could be deeply traumatic for some women. Postnatal PTSD is, thus, one of the lesser‐researched perinatal mood and anxiety disorders (PMAD). In 1997, the phenomenon was recognized widely enough that the first large‐scale study was carried out by a group in Sweden.^1^ They found that 1.6% of postnatal women studied showed symptoms of PTSD. A recent meta‐analysis found the prevalence of postnatal PTSD to be higher at 4.0% in the community, and 18.9% in at‐risk populations.^2^ Risk factors for postnatal PTSD include nulliparity, increased anxiety, feeling out of control, and lack of social support.^1^, ^3^ Research suggests that mode of birth is also a risk factor, particularly cesarean birth (CB).^4^, ^5^ This finding aligns with research on other PMADs, which suggests that both postnatal depression and anxiety are more common after CB.^6^, ^7^

In recent years, cesarean births have become much more common and, in some countries, for example, Brazil, a planned CB is the most common mode of birth in private health care settings.^8^ In low resource countries, such as those in sub‐Saharan Africa, the increase in the number of CBs performed in recent years reflects higher numbers of trained health care professionals available to perform the procedure in life‐threatening cases^9^; this is a positive shift that could help to reduce maternal and infant mortality and morbidity. However, in some low resource countries, as with the Dominican Republic (which has the highest CB rate in the world at 58.9%),^8^ most CBs are performed with no medical indication. The WHO estimates that when the CB rate increases above 15%, there is no further improvement to maternal and neonatal mortality rate, and adverse outcomes start to increase.^10^

Given the global increase in the rate of CB,^9^ an association between this mode of birth and postnatal PTSD could have wide implications. This review aimed to investigate whether postnatal PTSD is more common after CB or other modes of birth, to raise awareness of the risks of developing PTSD, and to encourage better recognition and treatment of the condition. Previous research has explored a relationship between CB and PTSD,^11^ and the psychosocial implications of emergency cesarean in particular.^12^ This mixed‐methods systematic review and meta‐analysis is the first to compare the impact of different modes of birth on maternal postnatal PTSD. For the purpose of this review, modes of birth were categorized as elective cesarean birth (ElCB), emergency cesarean birth (EmCB), spontaneous vaginal delivery (SVD), and instrumental vaginal delivery (IVD). IVD was classified as the use of forceps and/or vacuum extraction (ventouse).^13^

PRIMARY REVIEW QUESTIONS:

- Are women who have a CB more likely to experience PTSD postnatally than women who have a VD?
- What are the views of women who have postnatal PTSD on the impact of birth mode on their PTSD?
- What are the views of those who support women (i.e., family, peer support groups, and health care professionals) on the impact of birth mode on development of PTSD?

SECONDARY REVIEW QUESTIONS:

- Is an EmCB more likely to trigger symptoms and signs of PTSD than an ElCB?
- Is an IVD more likely to trigger symptoms and signs of PTSD than an SVD?
- Does a previous history of postnatal PTSD impact a woman's decision making about her mode of birth in subsequent pregnancies?
- How do individuals who have PTSD perceive the impact of social/peer support on their mental health outcomes?

## 2 METHOD

### 2.1 Eligibility criteria

This project included quantitative and qualitative studies to provide a holistic review of the literature. Studies from 1990 onwards were included, as research on postnatal PTSD commenced in this decade.^1^ Our review included studies, which explored PTSD in women who gave birth by different modes of birth as described in the introduction.

For quantitative research, studies of women who had given birth within 6 months before recruitment to a live, term, singleton infant in high resource countries were included.^14^ Qualitative studies were included if participants were individuals in the postnatal period who met the above criteria, or people who supported them. This category included partners, friends, family, peers, and health care professionals.

### 2.2 Search strategy

The review protocol was registered on the PROSPERO International Prospective Register of Systematic Reviews (CRD42018089132).^15^ Searches were conducted in CINAHL, the Cochrane Library, MEDLINE, PsycINFO, and Scopus on March 20, 2018, and update searches were performed on October 25, 2019, and February 11, 2021. Two searches were run in each database: one quantitative and one qualitative. Reference searching was performed in relevant reviews identified by the searches. Full search strategies are detailed in Appendices S1 and S2.

### 2.3 Study selection and data extraction

Abstract screening was undertaken followed by full‐text assessment by JC and Y‐SC. Papers that met eligibility criteria were included. When a paper did not explicitly state these criteria, authors were contacted via email

A data extraction form was created for quantitative and qualitative studies. From quantitative studies, the data extracted included incidence of postnatal PTSD for women who underwent different modes of birth in the form of odds ratios comparing the mode of birth studied with SVD where possible. For qualitative studies, key findings (i.e., themes) were extracted.

### 2.4 Quality appraisal

Quality was assessed independently by JC and Y‐SC using the Newcastle‐Ottawa scale (NOS)^16^ for quantitative cohort studies, the Centre for Evidence Based Medicine (CEBM) Critical Appraisal Checklist^17^ for cross‐sectional studies, and the Critical Appraisal Skills Programme (CASP) tool^18^ for qualitative studies. Any disagreements between the two reviewers were resolved through discussion.

### 2.5 Outcome measures

#### 2.5.1 Primary outcomes

The primary outcome of interest was PTSD status in the six‐month period after birth. The diagnosis of PTSD should have been based on DSM criteria, using the Impact of Event Scale (IES) or another validated scale used by study authors. Data on symptoms of PTSD, which did not meet full criteria for a diagnosis of PTSD, were also included, as not all study participants had undergone a full diagnostic interview. Qualitative data were included which described the attitudes toward and experiences of different modes of birth by individuals who developed postnatal PTSD and those who supported them.

#### 2.5.2 Secondary outcomes

Secondary outcomes included the impact of a history of postnatal PTSD on subsequent birth‐planning. Research indicates that individuals who experience a traumatic birth are more likely to request an elective cesarean,^19^ so studies investigating this relationship were also evaluated. We also evaluated how individuals with postnatal PTSD perceived the impact of social support on their mental health outcomes as a secondary outcome.

### 2.6 Data analysis

The data from the quantitative studies were assessed for suitability in a meta‐analysis. Studies were included if they reported sample size, mean PTSD score, and standard deviation at a relevant follow‐up point. Meta‐analyses were conducted to explore the effect of mode of birth on the score of PTSD symptoms. Because of the range of scales used, it was necessary to convert each to standardized mean difference (SMD) using Hedge's g^20^ as implemented in the Stata network suite of commands.^21^ When the reporting of subgroups differed, pooling was performed in line with recommendations from the Cochrane handbook.^22^ Results were interpreted using Cohen's guidelines for interpretation of SMD magnitude^23^; 0.2 is considered a small difference, 0.5 a medium difference, and 0.8 a large difference. Fixed‐effects models were used given the limitations of using random‐effects models with small numbers of studies.^24^, ^25^ Estimates were produced from both meta‐analyses (considering only studies with directly relevant data) and network meta‐analyses (including information from indirect comparisons when estimating the effect of interest). Heterogeneity was assessed through a comparison of the study characteristics. *I* ^2^ could not be calculated. Qualitative and quantitative studies not suitable for statistical analysis were reported narratively.

## 3 RESULTS

### 3.1 Search results

**FIGURE 1:**
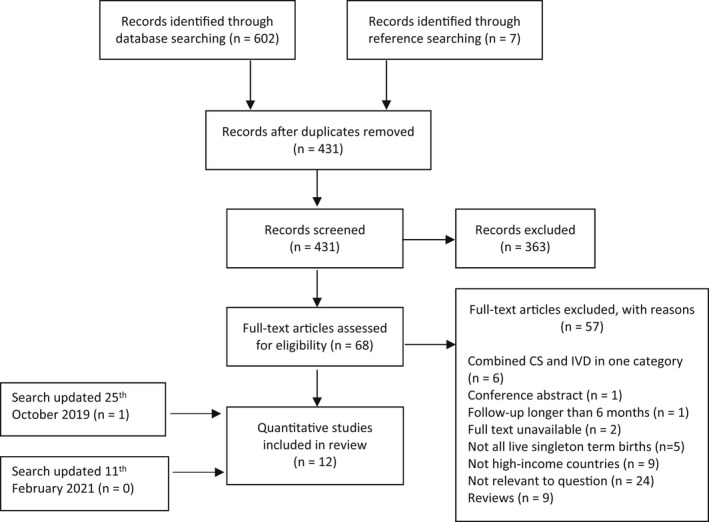
Preferred Reporting Items for Systematic Reviews and Meta‐Analyses (PRISMA) flow diagram of systematic search for quantitative studies

**FIGURE 2:**
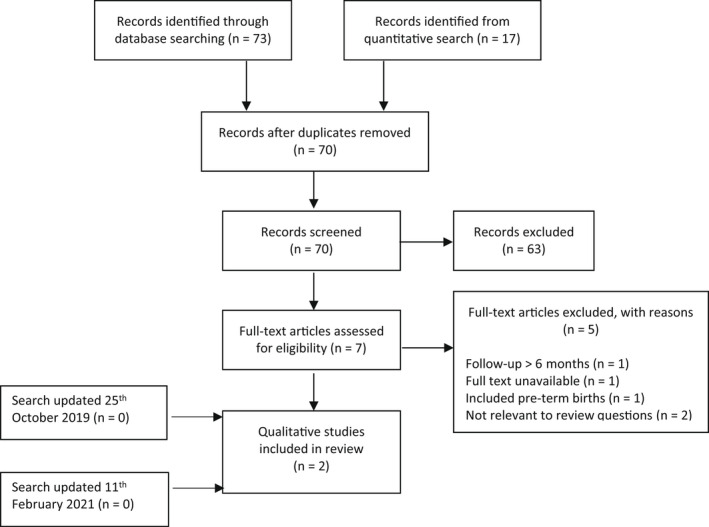
Preferred Reporting Items for Systematic Reviews and Meta‐Analyses (PRISMA) flow diagram of systematic search for qualitative studies

A systematic search on March 20, 2018 retrieved 602 quantitative studies from the five databases and an additional seven studies from reference searching of appropriate reviews identified by the search (Figure 1). After duplicates were removed, 431 records remained for abstract screening. Of these, full‐text screening was carried out on 68, after which 11 quantitative studies were included. An updated search on October 25, 2019, retrieved one additional relevant quantitative study, and the updated search on February 11, 2021, retrieved no more studies. A qualitative search on March 20, 2018, retrieved 73 studies from the five databases. Seventeen qualitative studies were found in the quantitative search process and added to the qualitative group (Figure 2). After duplicates were removed, 70 records remained for abstract screening. Full‐text analysis was performed on seven studies, of which two met the inclusion criteria. Updated searches on October 25, 2019, and February 11, 2021, retrieved no new qualitative studies.

### 3.2 Study characteristics

The 12 quantitative studies were all observational: 10 were cohort and two were cross‐sectional (Table 1).^26^, ^27^ Both qualitative studies presented findings from interviews (Table 2). In the quantitative group, sample sizes ranged from 42 to 1842, with a total of 5567 women. Sample sizes in the qualitative studies were much smaller, ranging from eight to 84, totaling 92 participants. Three quantitative studies were conducted in Canada, two in England, two in Israel, two in Sweden, one in Australia, one in Germany, and one in the United States. The two qualitative studies were both from Sweden. Recruitment of participants for the studies was mostly through community midwifery services or hospital maternity wards, but one recruited participants through the Internet and via maternity services^27^; one recruited specialist midwives from clinics caring for women with postnatal PTSD.^28^

While the qualitative studies used semi‐structured interviews, the quantitative studies all used diagnostic tools to examine the prevalence of PTSD among participants. Several diagnostic tools were used, with the most commonly used scales being the Post‐traumatic Stress Diagnostic Scale^29^ (used in three studies^27^, ^30^, ^31^) and the Impact of Event Scale^32^ (also used in three studies).^33^, ^34^, ^35^

**TABLE 1:** Summary of findings of quantitative search

Study	Intervention type	Study sample	Aims of study	Methodology	Outcome measures	Important results	Quality appraisal
Cohen, 2004^40^	SVD,^a^ IVD,^b^ CB^c^	N = 240	Discover the link between factors making for a difficult birth and postnatal PTSD symptoms.	Longitudinal. Questionnaire (phone).	Davidson Trauma Scale (DTS)	No significant difference between PTSD symptoms across 3 modes of birth (Chi‐squared *P* value 0.838).	NOS: Medium
Creedy, 2000^37^	SVD, forceps, vacuum, ElCB,^d^ EmCB,^e^	N = 592	Identify risk factors for psychological distress after childbirth.	Prospective longitudinal. Interview (phone).	Post‐traumatic Stress Symptoms interview (PSS)	EmCB, forceps and vacuum deliveries associated with PTSD symptoms (β = 0.196 *P* = 0.0001, β = 0.173 *P* = 0.0001 and β = 0.135 *P* = 0.003).	NOS: Medium
Dekel, 2019^26^	NAVD,^f^ SVD, IVD, ElCB, EmCB	N = 685	Ascertain whether women who undergo different modes of deliveries also differ in regard to their mental health postnatally.	Longitudinal. Questionnaire (internet).	PTSD checklist for DSM‐5 (PCL‐5)	PCL‐5 score EmCB> ElCB> IVD > SVD > NAVD. Delivery mode accounted for 13% of variance in symptoms severity (Pillai's T = 0.13, F[36, 2700] = 2.48, *P* < 0.0001).	CEBM: 6/12
Fairbrother, 2007^38^	VD,^g^ CB	N = 99	Examine psychological and obstetrical risk factors predicting postnatal PTSD symptoms.	Prospective longitudinal. Questionnaire.	PTSD Symptoms Scale Self‐Report (PSS‐SR)	Significant association between mode of birth (CB versus VD) and PTSD	NOS: Medium
Feeley, 2017^39^	VD, ElCB, EmCB	N = 298	Investigating PTSD symptoms in 2 high‐risk groups (Low birthweight infant who was admitted to a NICU, and EmCB) and 2 low‐risk groups (VD and ElCB).	Longitudinal. Questionnaire & interview (visited at home).	Perinatal PTSD Questionnaire (PPQ)	PPQ mean score at 5 weeks NICU > EmCB > VD > ElCB (*P* = 0.01). No significant difference between PPQ scores in clinical range at 5 weeks. EmCB did not have significantly greater PTSD symptoms than ElCB or VD.	NOS: Medium
Furuta, 2016^33^	SVD, IVD, ElCB, EmCB	N = 1824	Identify risk factors for PTSD in the postnatal period.	Secondary analysis of cohort study.	Impact of Event Scale (IES)	PTSD symptoms more common in IVD (*P* = 0.03 and 0.02 for symptoms of intrusion and avoidance, respectively) or EmCB (*P* < 0.001 for both intrusion and avoidance) compared with SVD.	NOS: Medium
Lyons, 1998^34^	SVD, forceps, ventouse, CB	N = 42	Investigate symptoms of PTSD in a group of first‐time mothers.	Longitudinal. Questionnaire (post).	IES	Mode of delivery was not related to PTSD symptoms (Kruskal–Wallis, H (3) = 2.39, *P* = 0.5).	NOS: Medium
Noyman‐Veksler, 2015^30^	SVD, EmCB, ElCB	N = 142	Investigate protective factors against PTSD symptoms after EmCB.	Longitudinal. Questionnaire.	PDS	No significant differences were found between the three modes of delivery.	NOS: Medium
Polachek, 2012^31^	SVD, IVD, ElCB, EmCB	N = 102	Examine postnatal PTSD in women in Israel and examine risk and protective factors.	Longitudinal. Questionnaire.	PDS	No significant association between mode of birth and PTSD.	NOS: Medium
Ryding, 1998^35^	SVD, IVD, ElCB, EmCB	N = 354	Compare psychological symptoms in women after EmCB, ElCB, IVD, and SVD.	Longitudinal. Questionnaire (post).	IES	EmCB > ElCB (*P* = 0.01), EmCB > SVD (*P* < 0.05).	NOS: Medium
Söderquist, 2009^36^	SVD, IVD, ElCB, EmCB	N = 508	Find risk factors in pregnancy for post‐traumatic stress (PTS) and depression 1 month after childbirth.	Longitudinal. Questionnaire.	Traumatic Event Scale (TES)	PTSD symptoms related to mode of delivery (F[1, 432] = 4.9, *P* = 0.002). EmCB led to more PTSD symptoms than SVD or ElCB (*P* = 0.01; *P* = 0.03, Scheffé post hoc test).	NOS: Medium
Vossbeck‐Elsebusch, 2014^27^	SVD, IVD, ElCB, EmCB, 2°CB^h^	N = 521	Investigate risk factors for PTSD outlined in Ehlers and Clark's (2000) model of PTSD.	Cross‐sectional. Questionnaire (online).	PDS (German version)	PDS scores differed depending on mode of birth, F(4,219) = 7.07, *P* < 0.001, ηp^2^ = 0.11 (univariate ANOVA). Post hoc Scheffé tests showed more symptoms in EmCB (M = 16.17, SD = 11.04, *p* = 0.001) than SVD (M = 7.04, SD = 7.56).	CEBM: 5/12

^a^ Spontaneous Vaginal Delivery.

^b^ Instrumental Vaginal Delivery.

^c^ Cesarean Birth.

^d^ Elective Cesarean Birth.

^e^ Emergency Cesarean Birth.

^f^ No Anesthesia Vaginal Delivery.

^g^ Vaginal Delivery.

^h^ Secondary cesarean birth (after onset of labor or after bursting of the amniotic sac).

**TABLE 2:** Summary of findings of qualitative search

Study	Question answered	Study sample	Aims of study	Methodology	Important results	Quality appraisal
Nyberg, 2010^28^	What are the views of those who support women (i.e., family and close friends, peer support groups, midwives, and other relevant health care staff) on impact of birth mode on development of PTSD?	N = 8 midwives at specialist clinic for women with PTSD after birth	Describe specialist midwives' experiences of working with women with postnatal PTSD.	Semi‐structured interviews analyzed using thematic content analysis.	Midwives reported a large number of women requesting ElCB after a previous traumatic birth, but felt that planning a vaginal delivery and appropriately supporting women throughout helped women confront their past experiences and move on from them. Women who did have ElCB tended to feel dissatisfied at their choice. Women felt like midwives were not supportive and did not listen to them properly and this worsened their experience of traumatic birth.	CASP: 9/10
Tham, 2010^43^	How do women who have PTSD perceive the impact of social/peer support on their mental health outcomes?	N = 84 women who had EmCB and developed PTSS (n = 42) or did not develop PTSS (n = 42)	Compare the experiences of women who underwent EmCB and the differences between those who did and did not develop PTSD.	Telephone interview 6 months after birth recorded by hand and analyzed by content analysis.	Women with PTSD were more likely to report: midwives seeming nervous, midwives being unsupported, not feeling involved in decisions about their treatment, and feeling like the baby would die.	CASP: 9/10

### 3.3 Quality assessment

The quality of the quantitative cohort studies was assessed using the NOS,^16^ which found that all 10 of the cohort studies had a medium risk of bias. The cross‐sectional studies were assessed using the CEBM checklist.^17^ One scored six out of a possible 12 desirable answers,^26^ and the other scored five,^27^ indicating the studies may not be of high quality. The qualitative studies were assessed using the relevant CASP checklist.^18^ Both achieved a score of at least 8 out of 10, suggesting the studies were of good quality. Full quality assessments can be seen in Appendices S6‐S8.

### 3.4 Description of findings

#### 3.4.1 Mode of birth and PTSD

Because of variation in study methodology and outcome measures, it was not possible to perform meta‐analyses across all studies for all the review questions. Categorization of mode of birth varied considerably and tended to be a secondary outcome in studies. Rather than reporting individual statistics, many studies simply performed an analysis of variance for all modes of birth.

For this reason, the only comparison possible across all studies was whether mode of birth was significantly associated with symptoms of PTSD. Overall, eight of the studies found significant variance in symptoms of PTSD across the modes of birth they investigated.^26^, ^27^, ^33^, ^35^, ^36^, ^37^, ^38^, ^39^ The remaining four studies did not find a significant association between mode of birth and PTSD.^30^, ^31^, ^34^, ^40^

#### 3.4.2 Cesarean versus vaginal delivery

**FIGURE 3:**
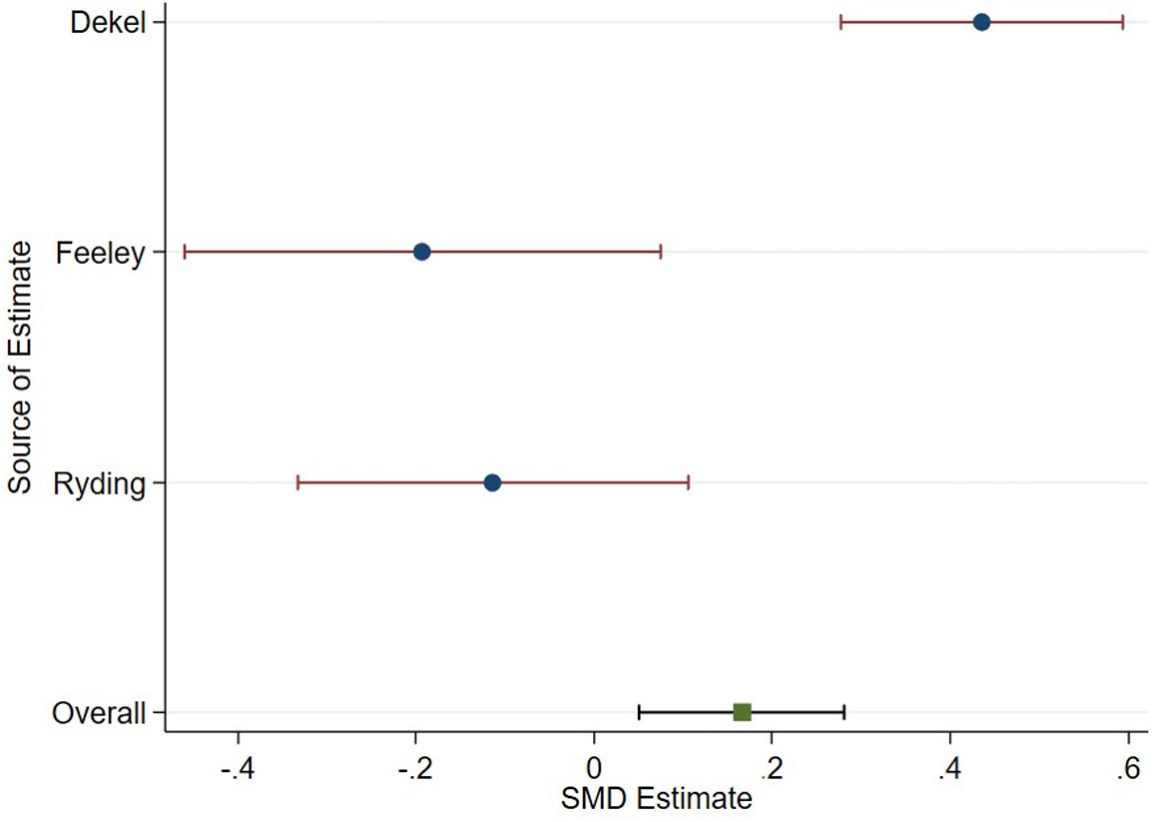
Meta‐Analysis of CS vs VD [Color figure can be viewed at wileyonlinelibrary.com]

Meta‐analysis of three studies^26^, ^35^, ^39^ showed that women who experienced a CB had a higher PTSD symptom score than women who had a VD (Figure 3). This difference (effect size = 0.17; 95% confidence interval = [0.05, 0.28]) was below that of the threshold to be considered small; however, it was statistically significant at the 0.05 threshold (*P* = 0.005). This may be a smaller effect size (ES) than expected, because this analysis pooled SVD and IVD, as well as ElCB and EmCB. Both poolings likely contain distinct groups which may have varying effects on PTSD.

#### 3.4.3 Emergency cesarean

Emergency cesarean birth is considered to be the most traumatic mode of birth, because of the stress associated with needing emergency surgery, often when a woman is in established labor.^1^, ^41^, ^42^ Six of the studies in the quantitative search found that EmCB was associated with an increased frequency of PTSD symptoms.^26^, ^27^, ^33^, ^35^, ^36^, ^37^

Meta‐analysis of three studies^26^, ^27^, ^35^ comparing EmCB to SVD suggested a medium difference (ES = 0.64; 95% CI = [0.48, 0.80]) of PTSD symptoms between these groups (Appendix S3) and was consistent with the network meta‐analyses (NMA) results (ES = 0.62; 95% CI = [0.47, 0.77]). EmCB was significantly worse, with the difference being the largest observed of any comparisons in this paper (*P* < 0.001).

Three studies^26^, ^35^, ^36^ found a significant difference in PTSD symptoms when comparing EmCB and ElCB, and another three found a difference that was not significant.^33^, ^37^, ^39^ A meta‐analysis of three studies^26^, ^35^, ^39^ showed a small‐to‐medium difference between EmCB and ElCB (ES = 0.36; 95% CI = [0.19, 0.54]) (Appendix S4), consistent with the NMA (ES = 0.40; 95% CI = [0.23, 0.57]). EmCB was associated with a higher score of PTSD symptoms—a statistically significant difference (*P* < 0.001).

With respect to the qualitative studies, Tham et al. interviewed 84 women who had an EmCB, 42 of whom went on to develop symptoms of PTSD.^43^ They found that women who developed PTSD symptoms after an EmCB were more likely to feel unsupported by staff and not feel involved in the decision to proceed to a cesarean relative to those who did not develop PTSD.

#### 3.4.4 Elective cesarean

Three of the quantitative studies that compared ElCB and EmCB found a significantly lower level of PTSD symptoms in the former.^26^, ^35^, ^36^ Nyberg et al. found that the specialist midwives they interviewed did not consider an elective cesarean to be a desirable mode of birth for women with PTSD from previous traumatic births, as those who proceeded with an ElCB in subsequent pregnancies often felt dissatisfied with their experience.^28^ The midwives believed that avoiding a vaginal birth for a subsequent delivery could leave women's fears of childbirth unresolved.

#### 3.4.5 Instrumental vaginal delivery

A meta‐analysis of two studies^26^, ^35^ found that IVD was associated with a higher PTSD score than SVD (ES = 0.38; 95% CI = [0.18, 0.59]) (Appendix S5), again consistent with the NMA (ES = 0.49; 95% CI = [0.29, 0.69]). This small‐to‐medium difference was highly statistically significant (*P* < 0.001).

Two studies subdivided instrumental vaginal deliveries into forceps and vacuum extraction deliveries.^34^, ^37^ One found statistically significant associations with PTSD symptoms for both forceps and vacuum extraction deliveries (β = 0.173 *P* = 0.0001; β = 0.135 *P* = 0.003, respectively).^37^ By contrast, the other did not find a significant relationship between PTSD symptoms and mode of delivery (Kruskal–Wallis, H (3) = 2.39, *P* = 0.5); however, the small study size meant it was unlikely any association would be detected if present.^34^

Five of the other studies combined instrumental vaginal births into one variable. Furuta et al. found PTSD symptoms were more common with IVD than SVD.^33^ Dekel et al. found greater PTSD symptoms in IVD than in SVD and their separate category of natural VD (without anesthesia).^26^ Ryding et al. did not directly compare IVD and SVD, but found no significant difference between PTSD symptoms in those who had IVD and those who had EmCB, which they found to be more closely associated with PTSD than both ElCB and SVD.^35^ Cohen et al. found no significant differences between IVD, SVD and CB,^40^ Polachek et al. did not find an association with any mode of birth and PTSD,^31^ and Vossbeck‐Elsebusch et al. found no statistically significant association between IVD and PTSD.^27^

## 4 DISCUSSION

### 4.1 Main findings

Although the studies identified by the quantitative search provided varied results, eight out of the 12 studies found that mode of birth was significantly associated with the development of symptoms of PTSD postnatally. Meta‐analysis showed that PTSD symptoms were more severe in women who had CBs compared to those who had VDs (*P* = 0.005). Symptoms were also significantly worse in women who had EmCB than in those who had either SVD (*P* < 0.001) or ElCB (*P* < 0.001). The significant difference between EmCB and ElCB may have confounded the first analysis, which pooled these two cesarean groups. Women who had cesarean births worried more about the safety of their baby and felt less satisfied overall with their birthing experiences,^44^, ^45^ a finding that aligns with the literature indicating that women may perceive CB as more traumatic than VD.^4^, ^5^

Each of the midwives interviewed by Nyberg et al. reported being asked by women with postnatal PTSD for an ElCB in a subsequent pregnancy but did not believe that this was the optimum mode of birth for this group of women.^28^ The other qualitative study included compared the experiences of women who did and did not develop symptoms of PTSD after an EmCB.^43^ Women who did not develop PTSD felt more supported by staff and more involved with the decision to proceed to a cesarean. This study suggests that although EmCB may be associated with an increased likelihood of postnatal PTSD, compassionate care and ensuring that women are involved in decision making can mediate against this effect. Research into parents' experiences showed that feeling listened to by health care professionals is a key component of a positive childbirth,^46^ reinforcing that the actions of health care professionals can improve outcomes for mental health postpartum.

As health care professionals closely involved with women pre‐, peri‐, and postpartum, midwives are in a unique position to help mitigate the risk of postnatal PTSD and other PMAD.^47^ One possible application of this is through midwifery‐led debriefing after traumatic birth; however, despite several randomized‐controlled trials reporting that women appreciate these debriefing sessions, a 2015 Cochrane review did not find high‐quality studies to support this, and concluded that more randomized‐controlled trials were needed.^48^

The impact of inadequate support from staff during labor and birth on women with PTSD supports the findings of Nyberg et al. This study reported that many women with postnatal PTSD told specialist midwives that they believed a lack of support and control during their birth had resulted in their PTSD.^28^ The issue of feeling in control during labor was identified by another Dutch study, which found that women who had a previous traumatic childbirth were more likely to have a positive experience in a subsequent birth if they felt in control during their birth.^49^

No studies were found that evaluated the role of support from partners, families, and peers as a potential mitigating factor against PTSD. This is an important avenue for future research.

### 4.2 Strengths and limitations

This is the first systematic review and meta‐analysis to combine information from small‐ and medium‐sized studies and present synthesized findings on the association between birth mode and maternal postnatal PTSD. However, there are several limitations. Not all studies included women with clinically diagnosed PTSD, and instead measured PTSD symptoms. There were also issues with the inclusion criterion of “live, term, singleton births.” Six of the included studies did not specify this within their papers, and only three replied to the authors of this review confirming that their paper met this criterion. This left three studies included on a benefit of the doubt basis.^27^, ^31^, ^34^ Another potential limitation was with the varying classifications of modes of birth by the different studies, which meant meta‐analysis was not possible across all 12 studies. It was also not possible to subdivide the EmCB category further according to urgency.

For this meta‐analysis, we compiled the most relevant clinical information, using robust statistical methods to synthesize the evidence on birthing methods and PTSD symptoms. A potential limitation was the assumption of equivalence in the measures used in the different studies and follow‐up points. A meta‐analysis of SMDs also assumes the populations of each study contain equivalent variation. The fixed‐effects model does not allow for any variation in the effect of birth mode on PTSD across studies. The violation of any of these assumptions may introduce bias into the estimates of effect or the uncertainty around them. The small number of studies means random‐effects models were unlikely to improve reliability.

### 4.3 Conclusions

Although there was variation among the studies, an overall conclusion can be made that mode of birth impacts PTSD in the postnatal period. Women who have emergency CB and instrumental VD might be at an increased risk, although it is important to note that women with all types of birth experiences can go on to develop PTSD, and this disorder is not limited to those who had a birth that would be deemed traumatic from a clinical perspective. Qualitative data indicated that although elective CB may not be associated with postnatal PTSD, they might have a negative impact on women with pre‐existing postnatal PTSD. Support and involvement in decision making from staff during labor may have a protective effect for women who experience emergency obstetric intervention, but more work must be done to confirm the above findings.

## Supporting information


Appendix S1–S8

## Data Availability

Data sharing is not applicable to this article as no new data were created or analyzed in this study.
