# Supplementary material for: Mode of birth and development of maternal postnatal post‐traumatic stress disorder: A mixed‐methods systematic review and meta‐analysis
Source: Birth. 2022 May 13;49(4):616–27. doi: 10.1111/birt.12649 (PMC9790679; doi:10.1111/birt.12649)
Supplement: Supplementary file 1 — Appendix S1–S8 [file BIRT-49-616-s001.docx]

**Supplementary information**

**Appendix 1: Quantitative Search Strategy**

**Cinahl:**

1. (MH Cesarean Section+)
2. Cesar?an* OR Caesar?an* OR C-section* OR C section* OR Surgical birth* OR Surgical deliver* OR Abdominal birth* OR Abdominal deliver*
3. S1 OR S2
4. Assisted vaginal deliver* OR Assisted vaginal birth* OR Operative vaginal deliver* OR Operative vaginal birth* OR Forceps OR Ventouse OR Obstetric intervention* OR Operative deliver* OR Operative birth* OR Instrumental deliver* OR Instrumental birth*
5. Spontaneous vaginal deliver* OR Spontaneous vaginal birth* OR Normal vaginal deliver* OR Normal vaginal birth* OR Normal deliver* OR Normal birth* OR Vaginal deliver* OR Vaginal birth* OR Non-operative deliver* OR Non-operative birth* OR Non-instrumental deliver* OR Non-instrumental birth* OR Natural deliver* OR Natural birth*
6. (MH "Stress Disorders, Post-Traumatic+")
7. PTSD OR Post?traumatic stress OR Posttraumatic stress OR Post traumatic stress OR Psychological trauma
8. S6 OR S7
9. S3 OR S4 OR S5
10. S8 AND S9
11. Limit: 1990 – 2019

**Cochrane**:

1. MeSH descriptor: [Cesarean Section] explode all trees
2. C*sar?an* OR C?section* OR "C section*" OR "Surgical birth*" OR "Surgical deliver*" OR "Abdominal birth*" OR "Abdominal deliver*"
3. "Assisted vaginal deliver*" OR "Assisted vaginal birth*" OR "Operative vaginal deliver*" OR "Operative vaginal birth*" OR Forceps OR Ventouse OR "Obstetric intervention*" OR "Operative deliver*" OR "Operative birth*" OR "Instrumental deliver*" OR "Instrumental birth*".mp.
4. "Spontaneous vaginal deliver*" OR "Spontaneous vaginal birth*" OR "Normal vaginal deliver*" OR "Normal vaginal birth*" OR "Normal deliver*" OR "Normal birth*" OR "Vaginal deliver*" OR "Vaginal birth*" OR "Non-operative deliver*" OR "Non-operative birth*" OR "Non-instrumental deliver*" OR "Non-instrumental birth*" OR "Natural deliver*" OR "Natural birth*"
5. MeSH descriptor: [Stress Disorders, Post-Traumatic] explode all trees
6. PTSD OR "Post?traumatic stress" OR "Posttraumatic stress" OR "Post traumatic stress" OR "Psychological trauma"
7. #1 OR #2 OR #3 OR #4
8. #5 OR #6
9. #7 AND #8
10. Limit: Publication Year from 1990 to 2019, in Trials

**Medline:**

1. Exp Cesarean Section/
2. C?esar?an*.mp.
3. C?section*.mp.
4. C section*.mp.
5. Surgical birth*.mp.
6. Surgical deliver*.mp.
7. Abdominal birth*.mp.
8. Abdominal deliver*.mp.
9. 1 or 2 or 3 or 4 or 5 or 6 or 7 or 8
10. Assisted vaginal deliver*.mp.
11. Assisted vaginal birth*.mp.
12. Operative vaginal deliver*.mp.
13. Operative vaginal birth*.mp.
14. Forceps.mp.
15. Ventouse.mp.
16. Obstetric intervention*.mp.
17. Operative deliver*.mp.
18. Operative birth*.mp.
19. Instrumental deliver*.mp.
20. Instrumental birth*.mp.
21. 10 or 11 or 12 or 13 or 14 or 15 or 16 or 17 or 18 or 19 or 20
22. Spontaneous vaginal deliver*.mp.
23. Spontaneous vaginal birth*.mp.
24. Normal vaginal deliver*.mp.
25. Normal vaginal birth*.mp.
26. Normal deliver*.mp.
27. Normal birth*.mp.
28. Vaginal deliver*.mp.
29. Vaginal birth*.mp.
30. Non-operative deliver*.mp.
31. Non-operative birth*.mp.
32. Non-instrumental deliver*.mp.
33. Non-instrumental birth*.mp.
34. Natural deliver*.mp.
35. Natural birth*.mp.
36. 22 or 23 or 24 or 25 or 26 or 27 or 28 or 29 or 30 or 31 or 32 or 33 or 34 or 35
37. Exp Stress Disorders, Post-Traumatic/
38. PTSD.mp.
39. Post?traumatic stress.mp.
40. Post traumatic stress.mp.
41. Psychological trauma.mp.
42. 37 or 38 or 39 or 40 or 41
43. 9 or 21 or 36
44. 42 AND 43
45. Limit: 1990 – current

**PsychINFO:**

1. Exp CESAREAN BIRTH/
2. C?esar?an*
3. C?section*
4. C section*
5. Surgical birth*
6. Surgical deliver*
7. Abdominal birth*
8. Abdominal deliver*
9. Or/1-8
10. Assisted vaginal deliver*
11. Assisted vaginal birth*
12. Operative vaginal deliver*
13. Operative vaginal birth*
14. Forceps
15. Ventouse
16. Obstetric intervention*
17. Operative deliver*
18. Operative birth*
19. Instrumental deliver*
20. Instrumental birth*
21. Or/10-20
22. Spontaneous vaginal deliver*
23. Spontaneous vaginal birth*
24. Normal vaginal deliver*
25. Normal vaginal birth*
26. Normal deliver*
27. Normal birth*
28. Vaginal deliver*
29. Vaginal birth*
30. Non-operative deliver*
31. Non-operative birth*
32. Non-instrumental deliver*
33. Non-instrumental birth*
34. Natural deliver*
35. Natural birth*
36. Or/22-35
37. Exp POSTTRAUMATIC STRESS DISORDER/
38. PTSD
39. Post?traumatic stress
40. Post traumatic stress
41. Psychological trauma
42. Or/37-41
43. 9 or 21 or 36
44. 42 AND 43
45. Limit: 1990 – current

**Scopus:**

TITLE-ABS-KEY ( c?esar?an*  OR  c?section*  OR  "C section*"  OR  "Surgical birth*"  OR  "Surgical deliver*"  OR  "Abdominal birth*"  OR  "Abdominal deliver*"  OR  "Assisted vaginal deliver*"  OR  "Assisted vaginal birth*"  OR  "Operative vaginal deliver*"  OR  "Operative vaginal birth*"  OR  forceps  OR  ventouse  OR  "Obstetric intervention*"  OR  "Operative deliver*"  OR  "Operative birth*"  OR  "Instrumental deliver*"  OR  "Instrumental birth*"  OR  "Spontaneous vaginal deliver*"  OR  "Spontaneous vaginal birth*"  OR  "Normal vaginal deliver*"  OR  "Normal vaginal birth*"  OR  "Normal deliver*"  OR  "Normal birth*"  OR  "Vaginal deliver*"  OR  "Vaginal birth*"  OR  "Non-operative deliver*"  OR  "Non-operative birth*"  OR  "Non-instrumental deliver*"  OR  "Non-instrumental birth*"  OR  "Natural deliver*"  OR  "Natural birth*" )  AND TITLE-ABS-KEY  ( ptsd  OR  "Post?traumatic stress"  OR  "Post traumatic stress"  OR  "Posttraumatic stress"  OR  "Psychological trauma" )  AND ( LIMIT-TO ( PUBYEAR ,  2019 )  OR  LIMIT-TO ( PUBYEAR ,  2018 )  OR  LIMIT-TO ( PUBYEAR ,  2017 )  OR  LIMIT-TO ( PUBYEAR ,  2016 )  OR  LIMIT-TO ( PUBYEAR ,  2015 )  OR  LIMIT-TO ( PUBYEAR ,  2014 )  OR  LIMIT-TO ( PUBYEAR ,  2013 )  OR  LIMIT-TO ( PUBYEAR ,  2012 )  OR  LIMIT-TO ( PUBYEAR ,  2011 )  OR  LIMIT-TO ( PUBYEAR ,  2010 )  OR  LIMIT-TO ( PUBYEAR ,  2009 )  OR  LIMIT-TO ( PUBYEAR ,  2008 )  OR  LIMIT-TO ( PUBYEAR ,  2007 )  OR  LIMIT-TO ( PUBYEAR ,  2006 )  OR  LIMIT-TO ( PUBYEAR ,  2005 )  OR  LIMIT-TO ( PUBYEAR ,  2004 )  OR  LIMIT-TO ( PUBYEAR ,  2003 )  OR  LIMIT-TO ( PUBYEAR ,  2002 )  OR  LIMIT-TO ( PUBYEAR ,  2001 )  OR  LIMIT-TO ( PUBYEAR ,  2000 )  OR  LIMIT-TO ( PUBYEAR ,  1999 )  OR  LIMIT-TO ( PUBYEAR ,  1998 )  OR  LIMIT-TO ( PUBYEAR ,  1997 )  OR  LIMIT-TO ( PUBYEAR ,  1996 )  OR  LIMIT-TO ( PUBYEAR ,  1995 )  OR  LIMIT-TO ( PUBYEAR ,  1994 )  OR  LIMIT-TO ( PUBYEAR ,  1993 )  OR  LIMIT-TO ( PUBYEAR ,  1992 )  OR  LIMIT-TO ( PUBYEAR ,  1991 )  OR  LIMIT-TO ( PUBYEAR ,  1990 ) )

**Appendix 2: Qualitative Search Strategy**

**Cinahl:**

1. (MH Cesarean Section+)
2. Cesar?an* OR Caesar?an* OR C-section* OR C section* OR Surgical birth* OR Surgical deliver* OR Abdominal birth* OR Abdominal deliver*
3. S1 OR S2
4. Assisted vaginal deliver* OR Assisted vaginal birth* OR Operative vaginal deliver* OR Operative vaginal birth* OR Forceps OR Ventouse OR Obstetric intervention* OR Operative deliver* OR Operative birth* OR Instrumental deliver* OR Instrumental birth*
5. Spontaneous vaginal deliver* OR Spontaneous vaginal birth* OR Normal vaginal deliver* OR Normal vaginal birth* OR Normal deliver* OR Normal birth* OR Vaginal deliver* OR Vaginal birth* OR Non-operative deliver* OR Non-operative birth* OR Non-instrumental deliver* OR Non-instrumental birth* OR Natural deliver* OR Natural birth*
6. S3 OR S4 OR S5
7. (MH "Stress Disorders, Post-Traumatic+")
8. PTSD OR Post?traumatic stress OR Posttraumatic stress OR Post traumatic stress OR Psychological trauma
9. S7 OR S8
10. Post?partum OR Post partum OR postpartum OR Post?natal OR Post natal OR Postnatal OR Peri?partum OR Peripartum OR Peri?natal OR Perinatal OR Puerperal
11. S9 AND S10
12. (MH "Attitude+")
13. (MH "Interviews+")
14. (MH "Qualitative Studies+")
15. (MH "Ethnographic Research")
16. (MH "Grounded Theory")
17. (MH "Thematic Analysis")
18. (MH "Content Analysis")
19. (MH "Observational Methods")
20. (MH "Constant Comparative Method”)
21. (MH "Field Notes")
22. (MH "Participant Observation")
23. (MH "Narratives")
24. (MH "Field Studies")
25. (MH "Audiorecording")
26. (MH "Focus Groups")
27. S12 OR S13 OR S13 OR S14 OR S15 OR S16 OR S17 OR S18 OR S19 OR S20 OR S21 OR S22 OR S23 OR S24 OR S25 OR S26
28. S6 AND S11 AND S27

**Cochrane:**

1. MeSH descriptor: [Cesarean Section] explode all trees
2. C*sar?an* OR C?section* OR "C section*" OR "Surgical birth*" OR "Surgical deliver*" OR "Abdominal birth*" OR "Abdominal deliver*"
3. "Assisted vaginal deliver*" OR "Assisted vaginal birth*" OR "Operative vaginal deliver*" OR "Operative vaginal birth*" OR Forceps OR Ventouse OR "Obstetric intervention*" OR "Operative deliver*" OR "Operative birth*" OR "Instrumental deliver*" OR "Instrumental birth*".mp.
4. "Spontaneous vaginal deliver*" OR "Spontaneous vaginal birth*" OR "Normal vaginal deliver*" OR "Normal vaginal birth*" OR "Normal deliver*" OR "Normal birth*" OR "Vaginal deliver*" OR "Vaginal birth*" OR "Non-operative deliver*" OR "Non-operative birth*" OR "Non-instrumental deliver*" OR "Non-instrumental birth*" OR "Natural deliver*" OR "Natural birth*"
5. MeSH descriptor: [Stress Disorders, Post-Traumatic] explode all trees
6. PTSD OR "Post?traumatic stress" OR "Posttraumatic stress" OR "Post traumatic stress" OR "Psychological trauma"
7. #1 OR #2 OR #3 OR #4
8. #5 OR #6
9. "Post?partum" OR "Post partum" OR "Post?natal" OR "Post natal" OR "Peri?partum" OR "Peri?natal" OR "Puerperal"
10. #8 AND #9
11. MeSH descriptor: [Qualitative Research] explode all trees
12. Interview/
13. view* OR experienc* OR opinion OR support* OR perspective OR perceive* OR perception* OR attitude* OR belie* OR feel* OR understand*
14. #11 or #12 or #13
15. #7 AND #10 AND #14
16. Limit: Publication Year from 1990 to 2019, in Trials

**Medline:**

1. Exp Cesarean Section/
2. C?esar?an*.mp.
3. C?section*.mp.
4. C section*.mp.
5. Surgical birth*.mp.
6. Surgical deliver*.mp.
7. Abdominal birth*.mp.
8. Abdominal deliver*.mp.
9. 1 or 2 or 3 or 4 or 5 or 6 or 7 or 8
10. Assisted vaginal deliver*.mp.
11. Assisted vaginal birth*.mp.
12. Operative vaginal deliver*.mp.
13. Operative vaginal birth*.mp.
14. Forceps.mp.
15. Ventouse.mp.
16. Obstetric intervention*.mp.
17. Operative deliver*.mp.
18. Operative birth*.mp.
19. Instrumental deliver*.mp.
20. Instrumental birth*.mp.
21. 10 or 11 or 12 or 13 or 14 or 15 or 16 or 17 or 18 or 19 or 20
22. Spontaneous vaginal deliver*.mp.
23. Spontaneous vaginal birth*.mp.
24. Normal vaginal deliver*.mp.
25. Normal vaginal birth*.mp.
26. Normal deliver*.mp.
27. Normal birth*.mp.
28. Vaginal deliver*.mp.
29. Vaginal birth*.mp.
30. Non-operative deliver*.mp.
31. Non-operative birth*.mp.
32. Non-instrumental deliver*.mp.
33. Non-instrumental birth*.mp.
34. Natural deliver*.mp.
35. Natural birth*.mp.
36. 22 or 23 or 24 or 25 or 26 or 27 or 28 or 29 or 30 or 31 or 32 or 33 or 34 or 35
37. Exp Stress Disorders, Post-Traumatic/
38. PTSD.mp.
39. Post?traumatic stress.mp.
40. Post traumatic stress.mp.
41. Psychological trauma.mp.
42. 37 or 38 or 39 or 40 or 41
43. Post?partum.mp.
44. Post partum.mp.
45. Post?natal.mp.
46. Post natal.mp.
47. Peri?partum.mp.
48. Peri?natal.mp.
49. Puerperal.mp.
50. 43 or 44 or 45 or 46 or 47 or 48 or 49 (Postnatal)
51. 42 AND 50
52. Qualitative Research/
53. Interview/
54. (theme$ or thematic).mp.
55. qualitative.af.
56. Nursing Methodology Research/
57. questionnaire$.mp.
58. ethnological research.mp.
59. ethnograph$.mp.
60. ethnonursing.af.
61. phenomenol$.af.
62. (grounded adj (theor$ or study or studies or research or analys?s)).af.
63. (life stor$ or women* stor$).mp.
64. (emic or etic or hermeneutic$ or heuristic$ or semiotic$).af. or (data adj1 saturat$).tw. or participant observ$.tw.
65. (social construct$ or (postmodern$ or post-structural$) or (post structural$ or poststructural$) or post modern$ or post-modern$ or feminis$ or interpret$).mp.
66. (action research or cooperative inquir$ or co operative inquir$ or co-operative inquir$).mp.
67. (humanistic or existential or experiential or paradigm$).mp.
68. (field adj (study or studies or research)).tw.
69. human science.tw.
70. biographical method.tw.
71. theoretical sampl$.af.
72. ((purpos$ adj4 sampl$) or (focus adj group$)).af.
73. (account or accounts or unstructured or openended or open ended or text$ or narrative$).mp.
74. (life world or life-world or conversation analys?s or personal experience$ or theoretical saturation).mp
75. (lived or life adj experience$).mp
76. cluster sampl$.mp.
77. observational method$.af.
78. content analysis.af.
79. (constant adj (comparative or comparison)).af.
80. ((discourse$ or discurs$) adj3 analys?s).tw.
81. narrative analys?s.af.
82. heidegger$.tw.
83. colaizzi$.tw.
84. spiegelberg$.tw.
85. (van adj manen$).tw.
86. (van adj kaam$).tw.
87. (merleau adj ponty$).tw.
88. husserl$.tw.
89. foucault$.tw.
90. (corbin$ adj2 strauss$).tw
91. glaser$.tw
92. 52 or 53 or 54 or 55 or 56 or 57 or 58 or 59 or 60 or 61 or 62 or 63 or 64 or 65 or 66 or 67 or 68 or 69 or 70 or 71 or 72 or 73 or 74 or 75 or 76 or 77 or 78 or 79 or 80 or 81 or 82 or 83 or 84 or 85 or 86 or 87 or 88 or 89 or 90 or 91
93. 9 or 21 or 36
94. 51 AND 92 AND 93

**PsychINFO:**

1. Exp CESAREAN BIRTH/
2. C?esar?an*
3. C?section*
4. C section*
5. Surgical birth*
6. Surgical deliver*
7. Abdominal birth*
8. Abdominal deliver*
9. Or/1-8
10. Assisted vaginal deliver*
11. Assisted vaginal birth*
12. Operative vaginal deliver*
13. Operative vaginal birth*
14. Forceps
15. Ventouse
16. Obstetric intervention*
17. Operative deliver*
18. Operative birth*
19. Instrumental deliver*
20. Instrumental birth*
21. Or/10-19
22. Spontaneous vaginal deliver*
23. Spontaneous vaginal birth*
24. Normal vaginal deliver*
25. Normal vaginal birth*
26. Normal deliver*
27. Normal birth*
28. Vaginal deliver*
29. Vaginal birth*
30. Non-operative deliver*
31. Non-operative birth*
32. Non-instrumental deliver*
33. Non-instrumental birth*
34. Natural deliver*
35. Natural birth*
36. Or/22-35
37. Exp POSTTRAUMATIC STRESS DISORDER/
38. PTSD
39. Post?traumatic stress
40. Post traumatic stress
41. Psychological trauma
42. Or/37-41 (PTSD)
43. Post?partum
44. Post partum
45. Post?natal
46. Post natal
47. Peri?partum
48. Peri?natal
49. Puerperal
50. Or/43-49 (Postnatal)
51. 42 AND 50
52. ((("semi-structured" or semistructured or unstructured or informal or "in-depth" or indepth or "face-to-face" or structured or guide or guides) adj3 (interview* or discussion* or questionnaire*)).ti,ab,id. or (focus group* or qualitative or ethnograph* or fieldwork or "field work" or "key informant")).ti,ab,id. or exp qualitative research/ or exp interviews/ or exp group discussion/ or qualitative study.md. not "Literature Review".md.
53. 9 or 21 or 36
54. 51 AND 52 AND 53

**Scopus:**

TITLE-ABS-KEY ( c?esar?an* OR c?section* OR "C section*" OR "Surgical birth*" OR "Surgical deliver*" OR "Abdominal birth*" OR "Abdominal deliver*" OR "Assisted vaginal deliver*" OR "Assisted vaginal birth*" OR "Operative vaginal deliver*" OR "Operative vaginal birth*" OR forceps OR ventouse OR "Obstetric intervention*" OR "Operative deliver*" OR "Operative birth*" OR "Instrumental deliver*" OR "Instrumental birth*" OR "Spontaneous vaginal deliver*" OR "Spontaneous vaginal birth*" OR "Normal vaginal deliver*" OR "Normal vaginal birth*" OR "Normal deliver*" OR "Normal birth*" OR "Vaginal deliver*" OR "Vaginal birth*" OR "Non-operative deliver*" OR "Non-operative birth*" OR "Non-instrumental deliver*" OR "Non-instrumental birth*" OR "Natural deliver*" OR "Natural birth*" ) AND TITLE-ABS-KEY ( Post?partum OR Postpartum OR"Post partum" OR Post?natal OR Postnatal OR "Post natal" OR Peri?partum OR Peripartum OR Peri?natal OR Perinatal OR Puerperal ) AND TITLE-ABS-KEY ( ptsd OR "Post?traumatic stress" OR "Post traumatic stress" OR "Posttraumatic stress" OR "Psychological trauma" ) AND TITLE-ABS-KEY(qualitative OR ethnol* OR ethnog* OR ethnonurs* OR emic OR etic OR leininger OR noblit OR "field note*" OR "field record*" OR fieldnote* OR "field stud*" or "participant observ*" OR "participant observation*" OR hermaneutic* OR phenomenolog* OR "lived experience*" OR heidegger* OR husserl* OR "merleau-pont*" OR colaizzi OR giorgi OR ricoeur OR spiegelberg OR "van kaam" OR "van manen" OR "grounded theory" OR "constant compar*" OR "theoretical sampl*" OR glaser AND strauss OR "content analy*" OR "thematic analy*" OR narrative* OR "unstructured categor*" OR "structured categor*" OR "unstructured interview*" OR "semi-structured interview*" OR "maximum variation*" OR snowball OR audio* OR tape* OR video* OR metasynthes* OR "meta-synthes*" OR metasummar* OR "meta-summar*" OR metastud* OR "meta-stud*" OR "meta-ethnograph*" OR metaethnog* OR "meta-narrative*" OR metanarrat* OR " meta-interpretation*" OR metainterpret* OR "qualitative meta-analy*" OR "qualitative metaanaly*" OR "qualitative metanaly*" OR "purposive sampl*" OR "action research" OR "focus group*" or photovoice or "photo voice" or "mixed method*" or view* or experienc* or opinion or support* or perspective or perceive* or perception* or attitude* or belie* or feel* or understand*) AND ( LIMIT-TO ( PUBYEAR, 2019 ) LIMIT-TO ( PUBYEAR,2018 ) OR LIMIT-TO ( PUBYEAR, 2017 ) OR LIMIT-TO ( PUBYEAR, 2016 ) OR LIMIT-TO ( PUBYEAR, 2015 ) OR LIMIT-TO ( PUBYEAR, 2014 ) OR LIMIT-TO ( PUBYEAR, 2013 ) OR LIMIT-TO ( PUBYEAR, 2012 ) OR LIMIT-TO ( PUBYEAR, 2011 ) OR LIMIT-TO ( PUBYEAR, 2010 ) OR LIMIT-TO ( PUBYEAR, 2009 ) OR LIMIT-TO ( PUBYEAR, 2008 ) OR LIMIT-TO ( PUBYEAR, 2007 ) OR LIMIT-TO ( PUBYEAR, 2006 ) OR LIMIT-TO ( PUBYEAR, 2005 ) OR LIMIT-TO ( PUBYEAR, 2004 ) OR LIMIT-TO ( PUBYEAR, 2003 ) OR LIMIT-TO ( PUBYEAR, 2002 ) OR LIMIT-TO ( PUBYEAR, 2001 ) OR LIMIT-TO ( PUBYEAR, 2000 ) OR LIMIT-TO ( PUBYEAR, 1999 ) OR LIMIT-TO ( PUBYEAR, 1998 ) OR LIMIT-TO ( PUBYEAR, 1997 ) OR LIMIT-TO ( PUBYEAR, 1996 ) OR LIMIT-TO ( PUBYEAR, 1995 ) OR LIMIT-TO ( PUBYEAR, 1994 ) OR LIMIT-TO ( PUBYEAR, 1993 ) OR LIMIT-TO ( PUBYEAR, 1992 ) OR LIMIT-TO ( PUBYEAR, 1991 ) OR LIMIT-TO ( PUBYEAR, 1990 ) )

**Appendix 3: EmCS vs SVD meta-analysis**

**
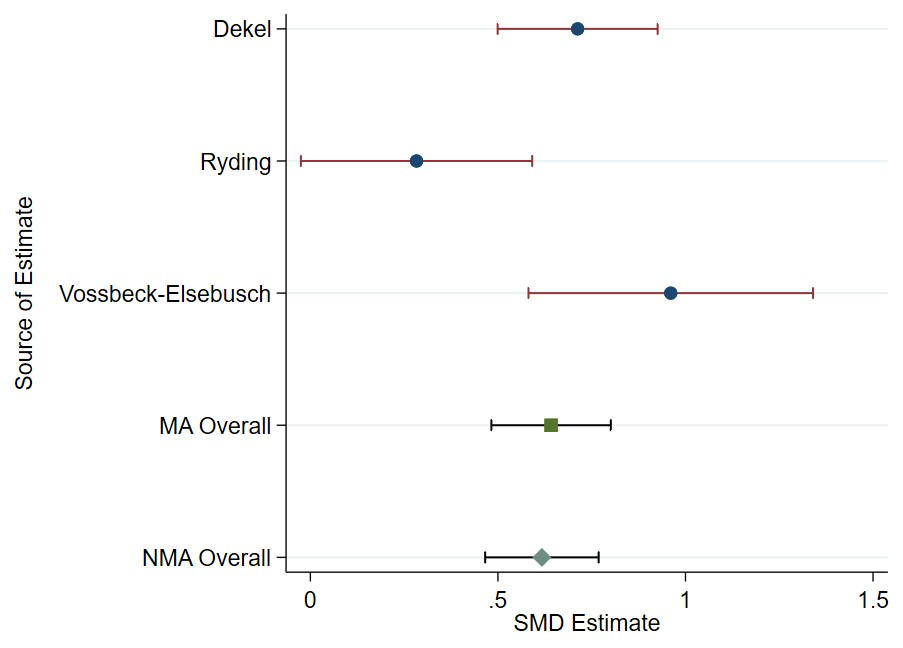
**

**Appendix 4: EmCS vs ElCS meta-analysis
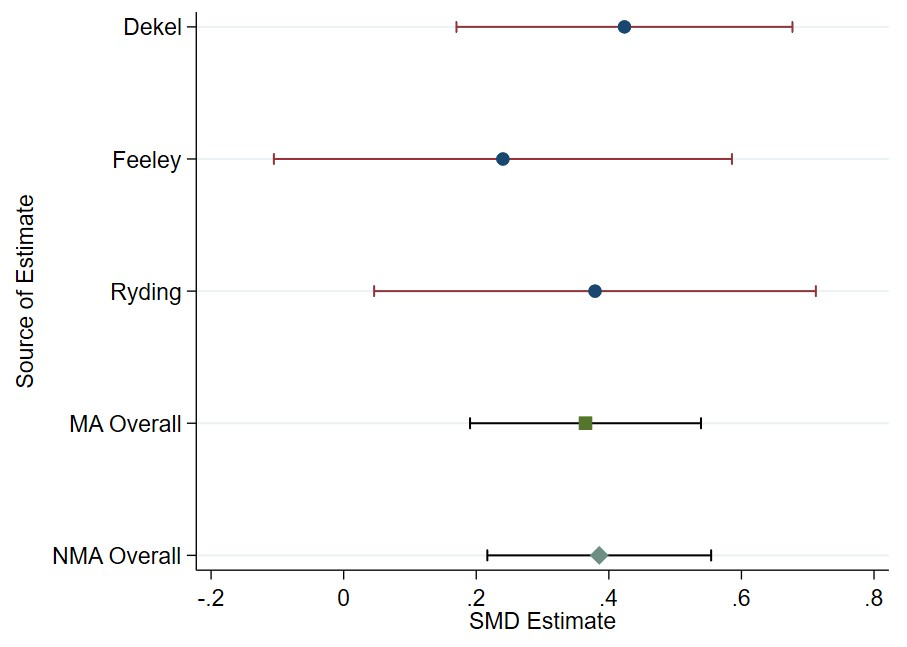
**

**Appendix 5: IVD vs SVD meta-analysis**

**
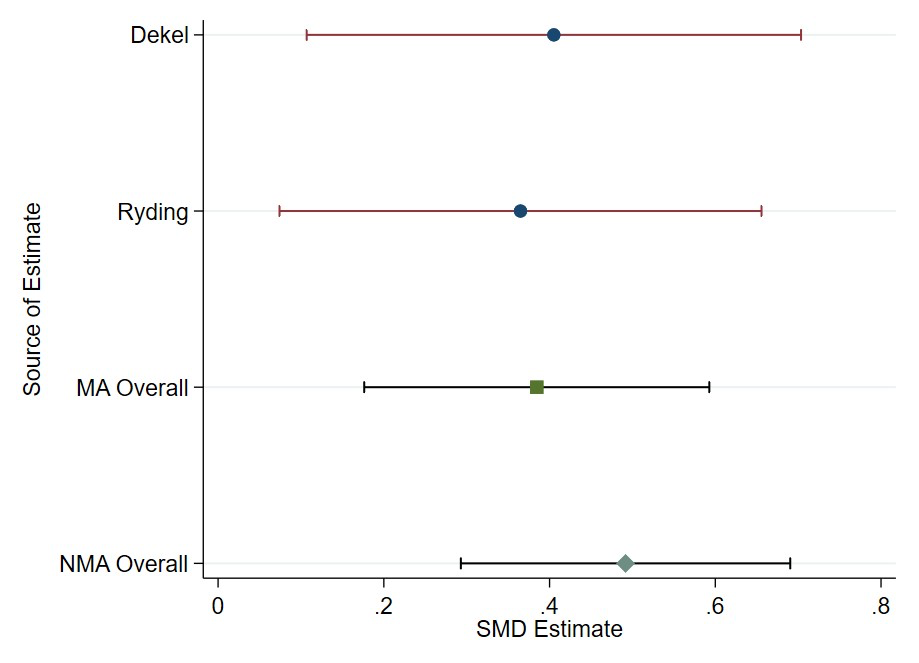
**

**Appendix 6: Quality assessment – Newcastle Ottowa Scale**

|  |  |  | Cohen, 2004 | Creedy, 2000 | Fairbrother, 2007 | Feeley, 2017 | Furuta, 2016 | Lyons, 1998 | Noyman-Veksler, 2015 | Polachek, 2012 | Ryding, 1998 | Söderquist, 2009 |
| --- | --- | --- | --- | --- | --- | --- | --- | --- | --- | --- | --- | --- |
| Selection | Representativeness of exposed cohort | Truly representative of the average postnatal woman in the community ☆ | x | x |  | x | x |  |  |  |  |  |
|  |  | Somewhat representative of the average postnatal woman in the community ☆ |  |  | x |  |  | x | x | x | x | x |
|  |  | Selected group of users |  |  |  |  |  |  |  |  |  |  |
|  |  | No description of the derivation of the cohort |  |  |  |  |  |  |  |  |  |  |
|  | Selection of non-exposed cohort | Drawn from the same community as the exposed cohort ☆ | x | x | x | x | x | x | x | x | x | x |
|  |  | Drawn from a different source |  |  |  |  |  |  |  |  |  |  |
|  |  | No description of the derivation of the non exposed cohort |  |  |  |  |  |  |  |  |  |  |
|  | Ascertainment of exposure | Secure record (eg surgical records) ☆ |  | x | x | x | x |  |  |  |  | x |
|  |  | Structured interview ☆ | x |  |  |  |  | x |  |  |  |  |
|  |  | Written self report |  |  |  |  |  |  |  |  |  |  |
|  |  | No description |  |  |  |  |  |  | x | x | x |  |
|  | Demonstration that outcome of interest was not present at start of study | Yes ☆ |  |  |  |  |  |  |  |  |  |  |
|  |  | No | x | x | x | x | x | x | x | x | x | x |
| Comparability | Comparability of cohorts on the basis of the design or analysis | Study controls for fear of childbirth ☆ |  |  |  |  |  |  |  |  |  |  |
|  |  | Study controls for any additional factor ☆ |  |  |  |  | x | x | x |  |  |  |
| Outcome | Assessment of outcome | Independent blind assessment ☆ |  |  |  |  |  |  |  |  |  |  |
|  |  | Record linkage ☆ |  |  |  |  |  |  |  |  |  |  |
|  |  | Self report | x | x | x | x | x | x | x | x | x | x |
|  |  | No description |  |  |  |  |  |  |  |  |  |  |
|  | Was follow-up long enough for outcomes to occur | Yes ☆ | x | x | x | x | x | x | x | x | x | x |
|  |  | No |  |  |  |  |  |  |  |  |  |  |
|  | Adequacy of follow up of cohorts | Complete follow up - all subjects accounted for ☆ |  |  |  |  |  |  |  |  |  |  |
|  |  | Subjects lost to follow up unlikely to introduce bias - <20% lost to follow up, or description provided of those lost ☆ | x |  |  | x | x | x | x | x | x | x |
|  |  | Lost > 20% to follow up and no description of those lost |  |  |  |  |  |  |  |  |  |  |
|  |  | No statement |  | x | x |  |  |  |  |  |  |  |

**Appendix 7: Quality assessment – Centre for Evidence-Based Management checklist**

|  | Dekel, 2019 | Vossbeck-Elsebusch, 2014 |
| --- | --- | --- |
| Did the study address a clearly focused question / issue? | Yes ☺ | Yes ☺ |
| Is the research method (study design) appropriate for answering the research question? | Yes ☺ | Yes ☺ |
| Is the method of selection of the subjects (employees, teams, divisions, organizations) clearly described? | Yes ☺ | Yes ☺ |
| Could the way the sample was obtained introduce (selection) bias? | Yes ☹ | Yes ☹ |
| Was the sample of subjects representative with regard to the population to which the findings will be referred? | No ☹ | No ☹ |
| Was the sample size based on pre-study considerations of statistical power? | No ☹ | No ☹ |
| Was a satisfactory response rate achieved? | Unclear | Unclear |
| Are the measurements (questionnaires) likely to be valid and reliable? | Yes ☺ | Yes ☺ |
| Was the statistical significance assessed? | Yes ☺ | Yes ☺ |
| Are confidence intervals given for the main results? | Yes ☺ | No ☹ |
| Could there be confounding factors that haven’t been accounted for? | Yes ☹ | Yes ☹ |
| Can the results be applied to your organization? | No ☹ | No ☹ |
| Total number of desirable answers (☺): | 6 | 5 |

**Appendix 8: Quality assessment – CASP Checklist**

| Study | Focused aim? | Qualitative method appropriate? | Research design appropriate? | Recruitment strategy appropriate? | Data collection method appropriate? | Relationship between researcher and participants considered? | Ethical issues considered? | Data analysis rigorous? | Results reported? | Results precise? | Results believable? | Results valuable? | Overall score |
| --- | --- | --- | --- | --- | --- | --- | --- | --- | --- | --- | --- | --- | --- |
| Nyberg, 2010 | Yes | Yes | Yes | Yes | Yes | No (no mention) | Yes | Yes | Yes | Yes | Yes | Yes | 9/10 |
| Tham, 2010 | Yes | Yes | Yes | Yes | No (recorded by hand, less accurate) | No (no mention) | Yes | Yes | Yes | Yes | Yes | Yes | 8/10 |
